# The lncRNA *RMST* is drastically downregulated in anaplastic thyroid carcinomas where exerts a tumor suppressor activity impairing epithelial-mesenchymal transition and stemness

**DOI:** 10.1038/s41420-023-01514-x

**Published:** 2023-07-01

**Authors:** Marco De Martino, Simona Pellecchia, Francesco Esposito, Federica Liotti, Sara Carmela Credendino, Nella Prevete, Myriam Decaussin-Petrucci, Paolo Chieffi, Gabriella De Vita, Rosa Marina Melillo, Alfredo Fusco, Pierlorenzo Pallante

**Affiliations:** 1 Istituto per l’Endocrinologia e l’Oncologia Sperimentale (IEOS) “G. Salvatore”, Consiglio Nazionale delle Ricerche (CNR), Via Pansini 5, 80131 Napoli, Italy; 2 Dipartimento di Medicina di Precisione, Università degli Studi della Campania “Luigi Vanvitelli”, Via De Crecchio 7, 80138 Napoli, Italy; 3 Dipartimento di Medicina Molecolare e Biotecnologie Mediche (DMMBM), Università degli Studi di Napoli “Federico II”, Via Pansini 5, 80131 Napoli, Italy; 4 Dipartimento di Scienze Mediche Traslazionali (DiSMeT), Università degli Studi di Napoli “Federico II”, Via Pansini 5, 80131 Napoli, Italy; 5 Service d’Anatomie et Cytologie Pathologiques, Centre de Biologie Sud, Groupement Hospitalier Lyon Sud, Universite Lyon 1, 69495 Pierre Bénite, France; 6 Instituto Nacional de Cancer, 37908, Laboratorio de Carcinogênese Molecular, Rua Andre Cavalcanti 37, Centro, 20231-050 Rio de Janeiro, Brazil

**Keywords:** Molecular biology, Cancer

## Abstract

Thyroid cancer is the most prevalent endocrine malignancy and comprises a wide range of lesions subdivided into differentiated (DTC) and undifferentiated thyroid cancer (UTC), mainly represented by the anaplastic thyroid carcinoma (ATC). This is one of the most lethal malignancies in humankind leading invariably to patient death in few months. Then, a better comprehension of the mechanisms underlying the development of ATC is required to set up new therapeutic approaches. Long non-coding RNAs (lncRNAs) are transcripts over 200 nucleotides in length that do not code for proteins. They show a strong regulatory function at both transcriptional and post-transcriptional level and are emerging as key players in regulating developmental processes. Their aberrant expression has been linked to several biological processes, including cancer, making them potential diagnostic and prognostic markers. We have recently analyzed the lncRNA expression profile in ATC through a microarray technique and have identified rhabdomyosarcoma 2-associated transcript (*RMST*) as one of the most downregulated lncRNA in ATC. *RMST* has been reported to be deregulated in a series of human cancers, to play an anti-oncogenic role in triple-negative breast cancer, and to modulate neurogenesis by interacting with SOX2. Therefore, these findings prompted us to investigate the role of *RMST* in ATC development. In this study we show that *RMST* levels are strongly decreased in ATC, but only slightly in DTC, indicating that the loss of this lncRNA could be related to the loss of the differentiation and high aggressiveness. We also report a concomitant increase of *SOX2* levels in the same subset of ATC, that inversely correlated with *RMST* levels, further supporting the *RMST/SOX2* relationship. Finally, functional studies demonstrate that the restoration of *RMST* in ATC cells reduces cell growth, migration and the stemness properties of ATC stem cells. In conclusion, these findings support a critical role of *RMST* downregulation in ATC development.

## Introduction

New hotspots in cancer research are represented by long non-coding RNAs (lncRNAs). LncRNAs, by interacting with DNA, RNA or proteins, serve as regulators at transcriptional and post-transcriptional levels. Their ability to impact on angiogenesis, proliferation, migration, apoptosis, and differentiation has been associated to both oncogenic and tumor suppressor functions. Furthermore, their presence in the peripheral blood and urine identified lncRNAs as candidate biomarkers in cancer diagnosis and prognosis [1].

Thyroid cancer (TC) represents the most common malignant endocrine tumor. Differentiated forms of thyroid cancer (DTC) comprise the common thyroid cancer types, papillary (PTC) and follicular (FTC), and present an excellent outcome in most cases. Conversely, anaplastic thyroid cancers (ATC) are the undifferentiated and most aggressive forms of TC. ATC is still a deadly cancer with an overall survival rate of 3–5 months after initial diagnosis [2].

Recent studies demonstrated that lncRNAs are deregulated in PTC, identifying several of them up- or down-regulated in PTC samples compared to controls [3]. LncRNAs exerting a tumor suppressor functions in PTC are, among the others, *NAMA* (noncoding RNA associated with MAP kinase pathway and growth arrest) that targets the MAPK signaling pathway [4], *PTCSC3* (papillary thyroid carcinoma susceptibility candidate 3) which predisposes to PTC and modulates the expression of several cancer-associated genes [5, 6], and *MPPED2-AS1*, whose loss of expression is associated with the onset of PTC [7]. LncRNA with tumor promoting functions [3] in PTC are, for example, *FAL1* (focally amplified long noncoding on chromosome 1) that is positively associated with risk of multifocality in PTC [8], and *NEAT1* (nuclear-enriched abundant transcript 1) that promotes tumor progression and is associated to increased tumor size in PTC [9].

To improve our comprehension of ATC pathogenesis, we have recently analyzed the lncRNA expression profile of nine ATC samples compared with five normal thyroid (NT) tissues through a microarray technique (Agilent Technologies, Santa Clara CA, USA) [10]. We found that the Prader Willi/Angelman region RNA5 (*PWAR5*) lncRNA was significantly downregulated in ATC compared to controls and exerts a tumor suppressor function being involved in proliferation and migration regulation [10, 11]. The same analysis allowed us to identify another lncRNA strongly downregulated in ATC, the rhabdomyosarcoma 2-associated transcript (*RMST*) [10]. *RMST* is a physiologic regulator of differentiation and stemness features of several tissues [12, 13], furthermore it has been described to be deregulated [14, 15] and to exert a critical tumor suppression role in cancer [16, 17].

Here, we confirm that *RMST* is one of the most downregulated lncRNAs in our set of thyroid carcinoma samples, in other public ATC set of samples (GSE65144 and GSE33630), and finally in a mouse model of inducible thyroid carcinogenesis. By performing functional analyses in culture cells, we demonstrate that *RMST* restoration in two ATC cell lines reduces cell growth, migration and invasion properties. Moreover, *RMST* overexpression reduces epithelial-mesenchymal transition (EMT) and stemness properties of ATC cells, as assessed by biochemical and functional assays.

Therefore, these findings support a critical role of *RMST* downregulation in ATC carcinogenesis.

## Results

### *RMST* is downregulated in ATC

To better investigate the role of *RMST* in TC progression, we searched for its expression levels in NT tissues compared to other normal tissue districts and in TC samples compared to NT.

**Fig. 1:**
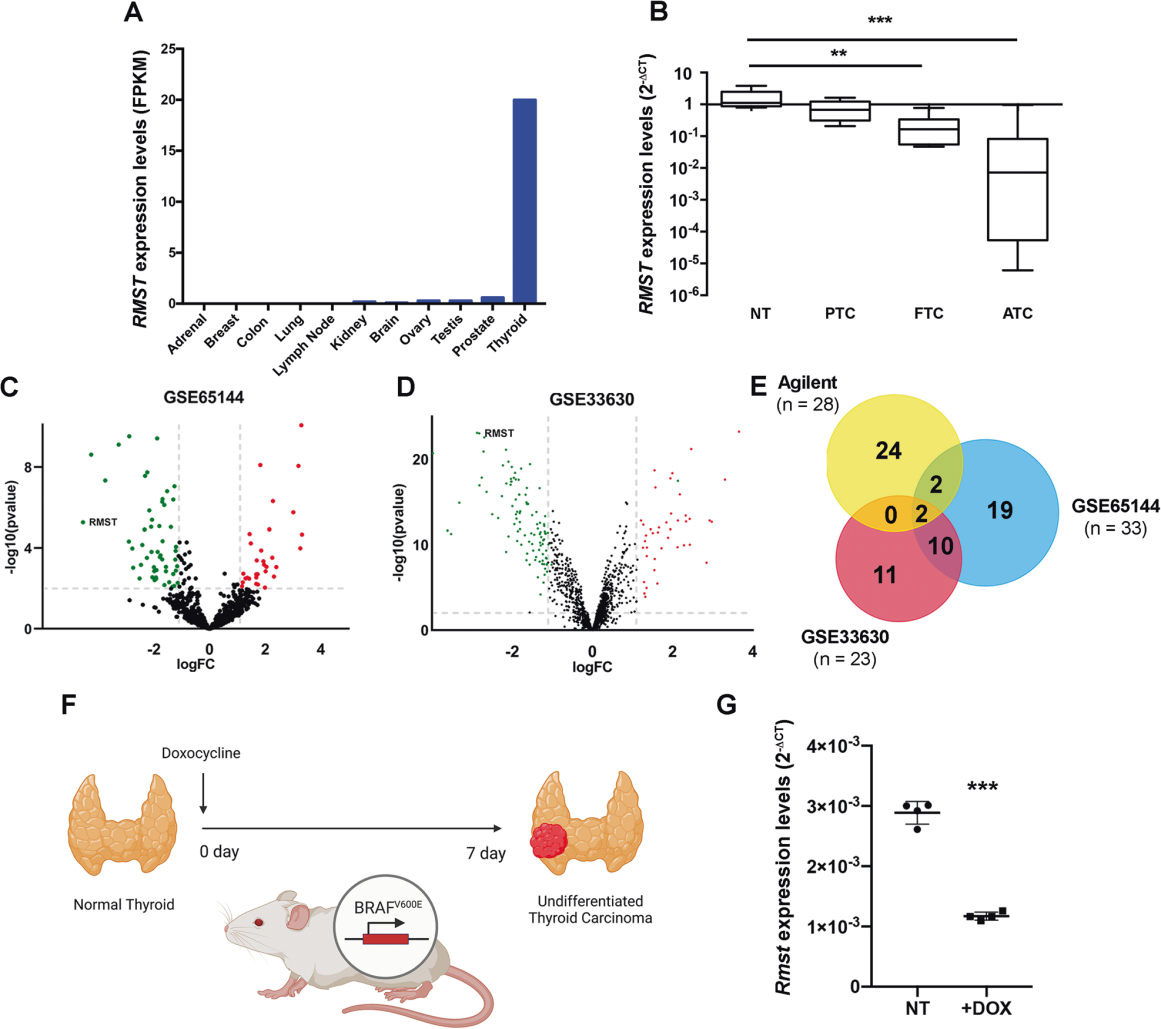
LncRNAs signature in ATC samples and NT tissues. **A** In silico analysis of *RMST* expression profile among human tissues from the CRlncRNA database (http://crlnc.liu-lab.com/rmst/). Data are reported as Fragment Per Kilobase Million (FPKM). **B** *RMST* levels were analyzed by qRT-PCR in a set of TC samples (PTC, *n* = 10; FTC, *n* = 6; ATC, *n* = 11) and NT (*n* = 5). Data are reported as 2^-ΔCt^ values ± SD. One-way analysis of variance (ANOVA) test, *p* < 0.05. **C**, **D** Volcano plots depict the differential expression of lncRNAs in GSE65144 and GSE33630 datasets. The *x*-axis is the log 2 fold change (FC) that corresponds to the mean expression value of each gene, while the *y*-axis is the -log10 (*p*-values) that indicates the level of significance of each gene. Each dot represents a gene: the red dots represent the most significant upregulated lncRNAs, whereas the green dots show the most significant downregulated lncRNAs in ATC *vs* NT. **E** Venn diagrams display the overlap of significantly ATC-downregulated lncRNAs in the Agilent array, in the GSE65144 and GSE33630 datasets. **F** Schematic representation of the induction of *BRAF*^V600E^-dependent TC following the administration of 2500 mg/kg doxycycline for 1 week (*n* = 4). Mice fed with normal food were used as control (NT) (*n* = 4). **G** qRT-PCR analysis of *Rmst* levels in a set of doxycycline-induced (+DOX) UTCs compared with the non-induced samples. Data are reported as 2^-ΔCt^ values ± SD.

Very interestingly, a bioinformatic analysis of *RMST* expression profile among human tissues from the CRlncRNA database (http://crlnc.liu-lab.com/rmst/), highlighted that *RMST* is mainly expressed in thyroid adult tissue (Fig. 1A) compared to the other districts, suggesting some specific role of *RMST* in thyroid physiology and differentiation.

We, then, evaluated its expression in a set of human TC specimens including 10 PTC, 6 FTC and 11 ATC by qRT-PCR in comparison with 5 NT. As shown in Fig. 1B, the *RMST* levels are strongly decreased in all the ATCs analyzed, whereas PTC and FTC show only a trend in the reduction of *RMST* levels (Fig. 1B). Thus, *RMST* downregulation appears correlated with the loss of the differentiated thyroid phenotype and with an increased aggressiveness of cancer samples. To further support these findings, we evaluated the differential lncRNA expression in the public GSE65144 and GSE33630 ATC microarray data downloaded from Gene Expression Omnibus (GEO) and based on Affymetrix Human Genome U133 Plus 2.0 Array. Interestingly, the Volcano plot analysis showed that *RMST* is one of the most and significantly downregulated lncRNAs in both microarray data (Fig. 1C, D). Then, by performing Venn diagrams on the data obtained from GSE65144, GSE33630 and our previously-published single channel Agilent array [10], we observed that *RMST* is one of the two most downregulated genes shared by the three analyzed arrays (Fig. 1E).

Then, in order to search for information on the clinical-pathological role of *RMST* in TC, we queried the cBioPortal for Cancer Genomics database (http://www.cbioportal.org) [18, 19]. Data on 500 TC samples revealed that *RMST* levels correlate with a better clinical outcome of TC patients as assessed by stratifying them for overall survival status, disease- and progression-free status (Supplementary Fig. 1).

Finally, we evaluated *Rmst* expression in a mouse model of undifferentiated TC (UTC) since *RMST* is a well-conserved gene throughout the evolution [20, 21]. We took advantage of the availability of the Tg-rtTA/TetO *BRAF*^V600E^ double transgenic mouse model (*BRAF*) that has the *BRAF*^V600E^ oncogene under the control of a modified tetracycline operator (TetO) and the thyroglobulin (Tg) promoter-controlled reverse tetracycline transactivator (rtTA) [22]. Doxycycline promotes *BRAF*^V600E^ expression leading to the rapid development of UTC, which has the same pathological characteristics of human ATC (Fig. 1F) [22]. As shown in Fig. 1G, the *Rmst* levels were strongly downregulated in the induced carcinomas (*n* = 4) compared with control thyroids (*n* = 4) (Fig. 1G).

### *RMST* levels inversely correlate to *SOX2* expression in human ATC and mouse UTC

*RMST* has been described as indispensable for physiologic neurogenesis. More in details, it interacts with SOX2, a transcription factor, thus regulating in a coordinated manner many downstream genes implicated in neurogenesis [23]. In the cancer context, SOX2 is a well-characterized key factor essential for cancer stem cell (CSC) self-renewal and reprogramming [24]. SOX2 has been reported also as an ATC stemness marker [25, 26] and as strongly involved in thyroid CSC-dependent chemoresistance [27].

**Fig. 2:**
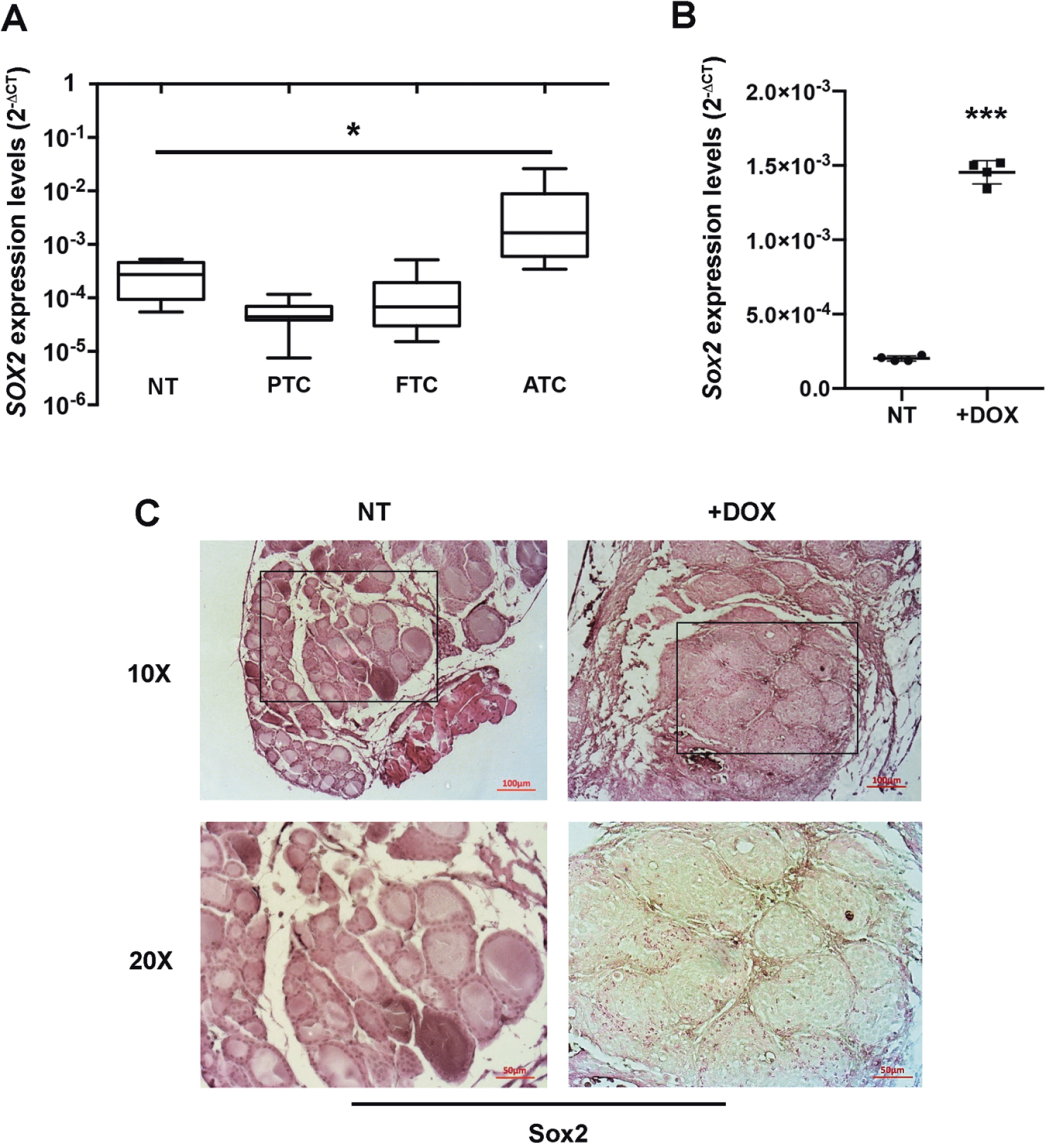
*SOX2* expression is inversely correlated to *RMST* levels. **A** *SOX2* levels were analyzed by qRT-PCR in the same set of TC samples reported in Fig. 1B. Data are reported as 2^-ΔCt^ values ± SD. One-way analysis of variance (ANOVA) test, *p* < 0.05. **B** qRT-PCR analysis of *Sox2* levels in a set of doxycycline-induced (+DOX) UTCs compared with the non-induced samples. Data are reported as 2^-ΔCt^ values ± SD. **C** Thyroid samples were fixed and paraffin-embedded, then sections were analyzed by immunohistochemical staining of Sox2. Eosin staining was then performed. Magnification: 10 × (upper panel) and 20 × (lower panel) of the 10 × boxed area. The images are representative of six different images.

We looked for *SOX2* expression levels in undifferentiated human and mouse thyroid lesions and analyzed them in relation to the *RMST* levels in the experimental systems above described (Fig. 1). As reported in Fig. 2A, the analysis of our set of samples revealed that *SOX2* mRNA levels were significantly upregulated in ATC samples compared to those observed in PTC, FTC and NT (Fig. 2A). The trend of *SOX2* expression in human TC samples is inverted compared to that of *RMST* in the same set of samples (Fig. 1B).

Consistently, *Sox2* expression levels were significantly higher in the mice UTC (induced through doxycycline, *n* = 4) samples compared with the control tissues (*n* = 4) (Fig. 2B). These findings were also confirmed at protein levels by an IHC analyses, where Sox2 signal is strongly increased in the induced UTCs compared with the thyroids derived from non-induced mice (Fig. 2C).

### Restoration of *RMST* expression impairs cell growth of ATC cells

Our expression data on both human and mouse samples indicate that *RMST* is strongly downregulated in undifferentiated thyroid lesions, and further support the hypothesis of a critical role of *RMST* downregulation in stemness and in progression of ATC.

**Fig. 3:**
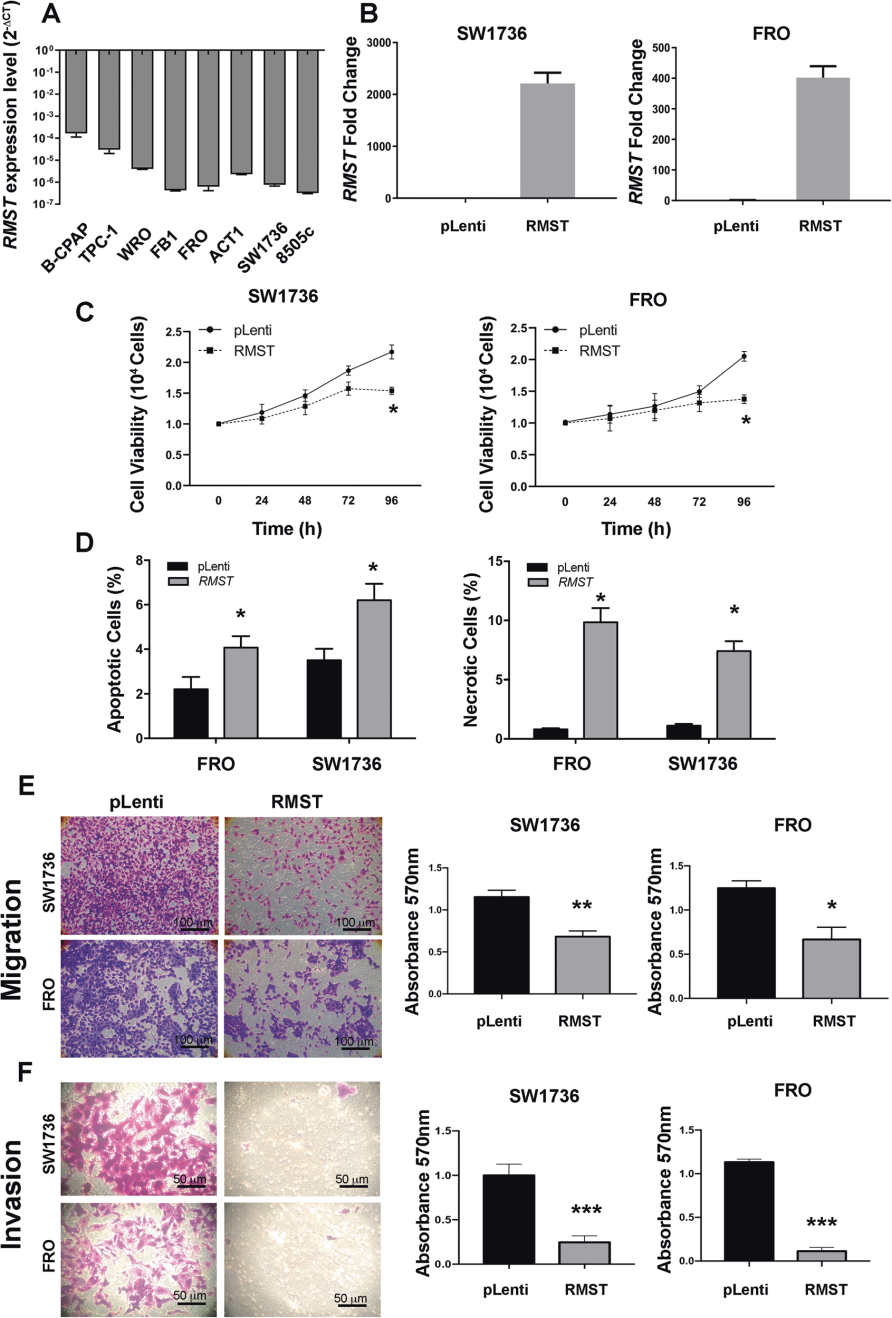
*RMST* overexpression impairs cell growth properties of ATC-derived cellular models. **A** qRT-PCR analysis of *RMST* levels in a set of human TC cell lines. Data are reported as 2^-ΔCt^ values ± SD. **B** qRT-PCR analysis of SW1736 and FRO cell lines stably expressing *RMST* compared to the relative empty vector (pLenti) transfected cells. Data were compared to pLenti, set equal to 1, and reported as 2^-ΔΔCt^ values ± SD. **C** Cell growth analysis of SW1736 and FRO cells stably expressing *RMST* or transfected with the relative control vector (pLenti). Cells were counted at 24, 48, 72, and 96 h after seeding. Values were obtained from three independent experiments. Data are reported as mean ± SD. Two-way ANOVA test (Bonferroni post-test: *RMST vs* pLenti, 96 h, *p < 0.05 in FRO cell line; *RMST vs* pLenti, 96 h, *p < 0.05 in SW1736 cell line). **D** AnxV/PI staining of SW1736 and FRO cells stably expressing *RMST* or transfected with the relative control vector (pLenti) after 18 h of serum deprivation. Apoptotic cells were AnxV^+^PI^-^, whereas necrotic^/^dead cells were AnxV^-^PI^+^ or AnxV^+^PI^+^. Data are represented as mean ± SD of three independent determinations on three mass populations of transfected cells, *p < 0.05 compared with the control cells (pLenti) by Student’s *t*-test. **E** Migrating cells were fixed with crystal violet solution (left panel) and the migration was quantified by reading absorbance at 570 nm (right panel) after elution in SW1736 and FRO cells. Values are reported as mean value ± SD, compared to the control vector pLenti, set equal to 1. Unpaired *t*-test: *RMST vs* pLenti, **p < 0.01 in SW1736 cells; *RMST vs p*Lenti, *p < 0.05 in FRO cells. Representative images of migration assays performed in SW1736 and FRO cells stably expressing *RMST* or carrying the corresponding pLenti vector are presented in the left panel (magnification 50 ×). **F** Invading cells were fixed with crystal violet solution (left panel) and the invasion was quantified by reading absorbance at 570 nm (right panel) after elution in SW1736 and FRO cells. Values are reported as mean value ± SD, compared to the pLenti. Unpaired *t*-test: *RMST vs* pLenti, ***p < 0.001 in SW1736 cells; *RMST vs* pLenti, ***p < 0.001 in FRO cells. Representative images of invasion assays performed in SW1736 and FRO cells stably transfected with *RMST* or the corresponding pLenti control vector are presented in the left panel (magnification 100 ×).

In order to investigate the *RMST* role in ATC from a functional point of view, we first of all evaluated by qRT-PCR its expression levels in a panel of PTC-derived (B-CPAP and TPC-1), FTC-derived (WRO) and ATC-derived (FB1, FRO, ACT1, SW1736 and 8505c) cell lines, including in our analysis as a control the Nthy-ori, an immortalized cell line derived from NT epithelial cells [28]. As shown in Fig. 3A, all TC cell lines (FB1, FRO, ACT1, SW1736 and 8505c) derived from ATC showed extremely reduced *RMST* expression levels compared to Nthy-ori (Fig. 3A). Then, we restored *RMST* expression in two ATC cell lines, FRO and SW1736, by using a pLenti-GIII-CMV-*RMST* (pLenti-*RMST*) vector (Fig. 3B). FRO and SW1736 ATC cells overexpressing *RMST* and the relative controls were characterized for their growth rate. We observed that both FRO and SW1736 cells transfected with pLenti-*RMST* showed a significant reduction in growth rate when compared with those carrying the corresponding empty vector (pLenti) (Fig. 3C).

We then assessed the effects of *RMST* on cell cycle and apoptosis in order to understand how it inhibits the proliferation of TC cells. We analyzed the cell cycle distribution in serum-deprived *RMST*-transfected cells compared to the corresponding empty vector control (pLenti) cells. Supplementary Fig. 2 shows that both FRO- and SW1736-*RMST* cells did not display differences in cell cycle phases compartmentalization compared to the relative pLenti-TC cells (Supplementary Fig. 2). Subsequently, to assess whether *RMST* could impact on TC cell growth by affecting their death rate we performed an AnxV/PI staining of *RMST*- and pLenti-TC cells. *RMST* transfection significantly increased the TC cell apoptotic and necrotic rate in basal conditions compared to controls as assessed by the percentage of AnxV^+^PI^-^ (apoptotic) and AnxV^-^PI^+^ or AnxV^+^PI^+^ (necrotic/dead) cells (Fig. 3D).

Thereafter, to further investigate whether *RMST* affects the malignant phenotype, we evaluated its role on the migration process of ATC cells. To this aim, migration ability was evaluated by employing Transwell assay on the *RMST* overexpressing FRO and SW1736 cells. As shown in Fig. 3E, the restoration of *RMST* expression significantly inhibited the migration properties of FRO and SW1736 cells in comparison to the control cells (Fig. 3E). Similar results were obtained in an invasion assay using the Transwell chambers coated with Matrigel, a reconstituted basement membrane. Fig. 3F shows that the invasion properties in both the *RMST*-overexpressing FRO and SW1736 cells were drastically reduced in comparison with the control cells (Fig. 3F).

### *RMST* regulates epithelial-mesenchymal transition and invasion in ATC cells

Epithelial-mesenchymal transition (EMT) represents the main program activated in cancer cells to ensure the acquisition of an aggressive phenotype [29]. Since the drastic *RMST* downregulation was associated with the extremely aggressive ATC phenotype, we hypothesized its involvement in the EMT programs.

**Fig. 4:**
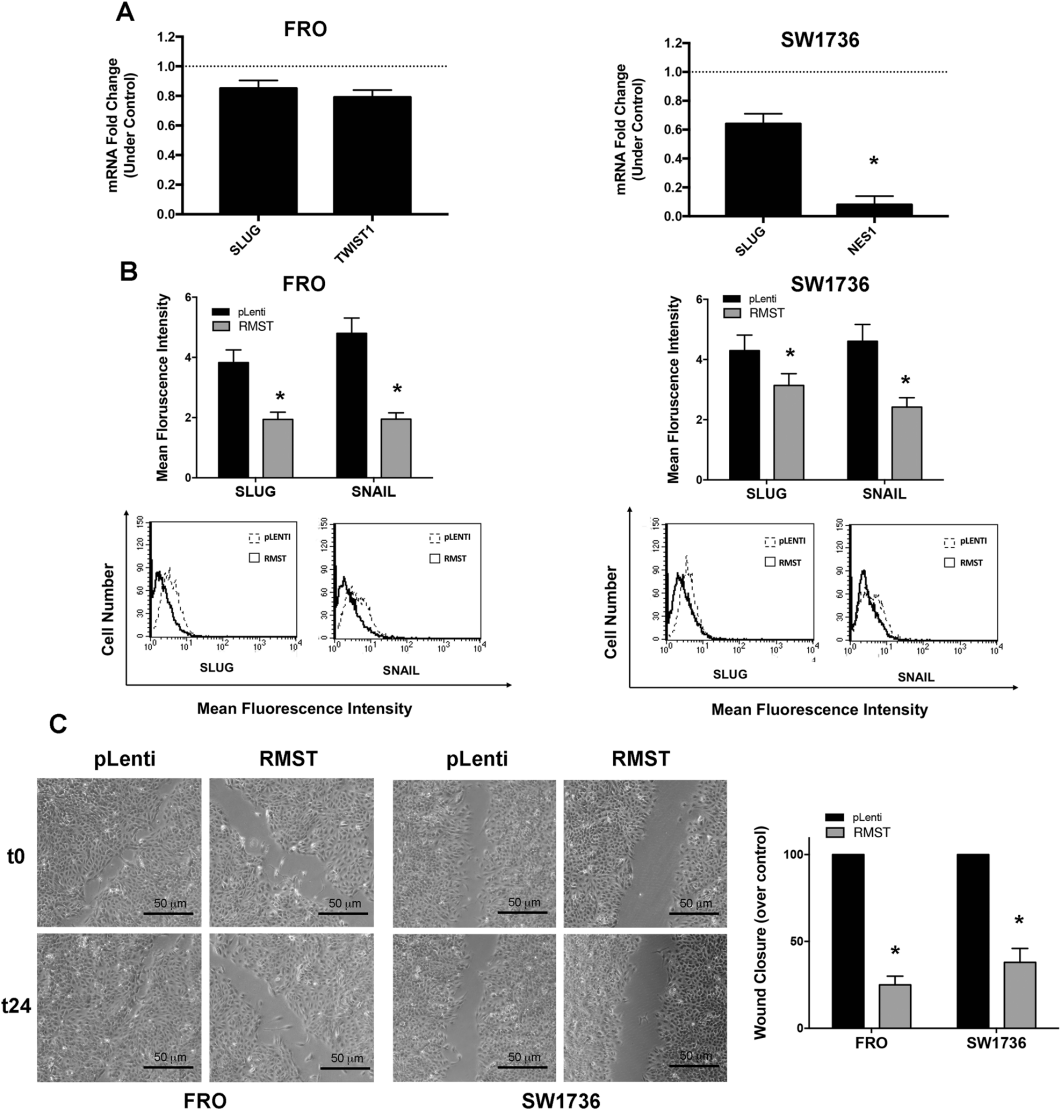
*RMST* overexpression dampens epithelial-mesenchymal transition in ATC cell lines. **A** qRT-PCR analysis showing the expression levels of *SLUG* and *TWIST1* in FRO-*RMST* compared to FRO-pLenti (left panel), and *SLUG* and *NES1* in SW1736-*RMST* compared to SW1736-pLenti (right panel). Data were compared to contol cells (pLenti), set equal to 1, and reported as 2^-ΔΔCt^ values ± SD. **B** Cytofluorimetric assay was employed to evaluate the expression of SLUG and SNAIL proteins in FRO-*RMST* (left panel) and SW1736-*RMST* (right panel) cells, compared to the respective control FRO-pLenti and SW1736-pLenti cells. Data are reported as Mean Fluorescence Intensity values ± SD of three independent determinations on three mass populations, *p < 0.05 compared to the control cells (pLenti) by Student’s *t*-test. Representative histogram plots are also presented. **C** Wound-healing assays of FRO-*RMST* and SW1736-*RMST* cells, compared to the respective FRO-pLenti and SW1736-pLenti control cells, in 10% of fetal bovine serum. Quantifications of wound closure (right panel) are shown together with photograms of migration (left panel; magnification 50 ×). The wound closure rate was calculated measuring the difference in wound width (WW) at time zero (t0) and after 24 h (t24). The formula used is as follows: wound closure rate = (WWt0-WWt24) *RMST*/(WWt0-WWt24) pLenti × 100. Values represent the average of triplicate experiments ± SD, *p < 0.05, compared to the control cells (pLenti) by Student’s *t*-test.

To evaluate biochemical and functional impact of *RMST* on EMT in ATC cells we analyzed the expression of master regulators of EMT and tested the invasion properties of the ATC cells overexpressing *RMST*. As reported in Fig. 4A, the transfection of *RMST* was able to sustain a trend in mRNA downregulation of several EMT markers (*SLUG, TWIST1, NES1*) in both FRO and SW1736 cell lines, being only *NES1* levels significantly downregulated in SW1736-*RMST* cells, compared to that transfected with the empty vector. Consistently, the FACS analysis performed on permeabilized TC cells transfected with *RMST* revealed a significant reduction in the protein levels of both SLUG and SNAIL, two critical EMT master regulators [29], compared to controls (Fig. 4B).

The invasiveness of tumor cells represents the functional phenotype of the EMT program together with the acquisition by epithelial cells of a fibroblast-like morphology [29]. Thus, to evaluate *RMST* effects on functional EMT, we first of all evaluated the morphology of FRO and SW1736 cells transfected with the empty vector or with *RMST*. Supplementary Fig. 3 shows that empty vector TC cells are spread and display a fibroblast-like morphology, reminiscent of EMT. These characteristics are significantly less evident in *RMST*-transfected cells, looking well adherent one to the other and with a classic epithelial morphology (Supplementary Fig. 3). To verify whether thyroid cells transfected with *RMST* reduced their response to functional EMT, a wound-healing assay was performed. In comparison to pLenti-TC cells, *RMST* cells less effectively triggered wound closure in culture medium completed with 10% fetal bovine serum, as displayed in Fig. 4C. Wound closure rate was 25 ± 5 and 38 ± 8 in FRO- and SW1736-*RMST* cells, respectively, compared to each FRO- and SW1736-pLenti cells, in which wound closure rate was considered equal to 100 (*p* < 0.05) (Fig. 4C).

### *RMST* negatively affects the stemness properties of ATC cells

**Fig. 5:**
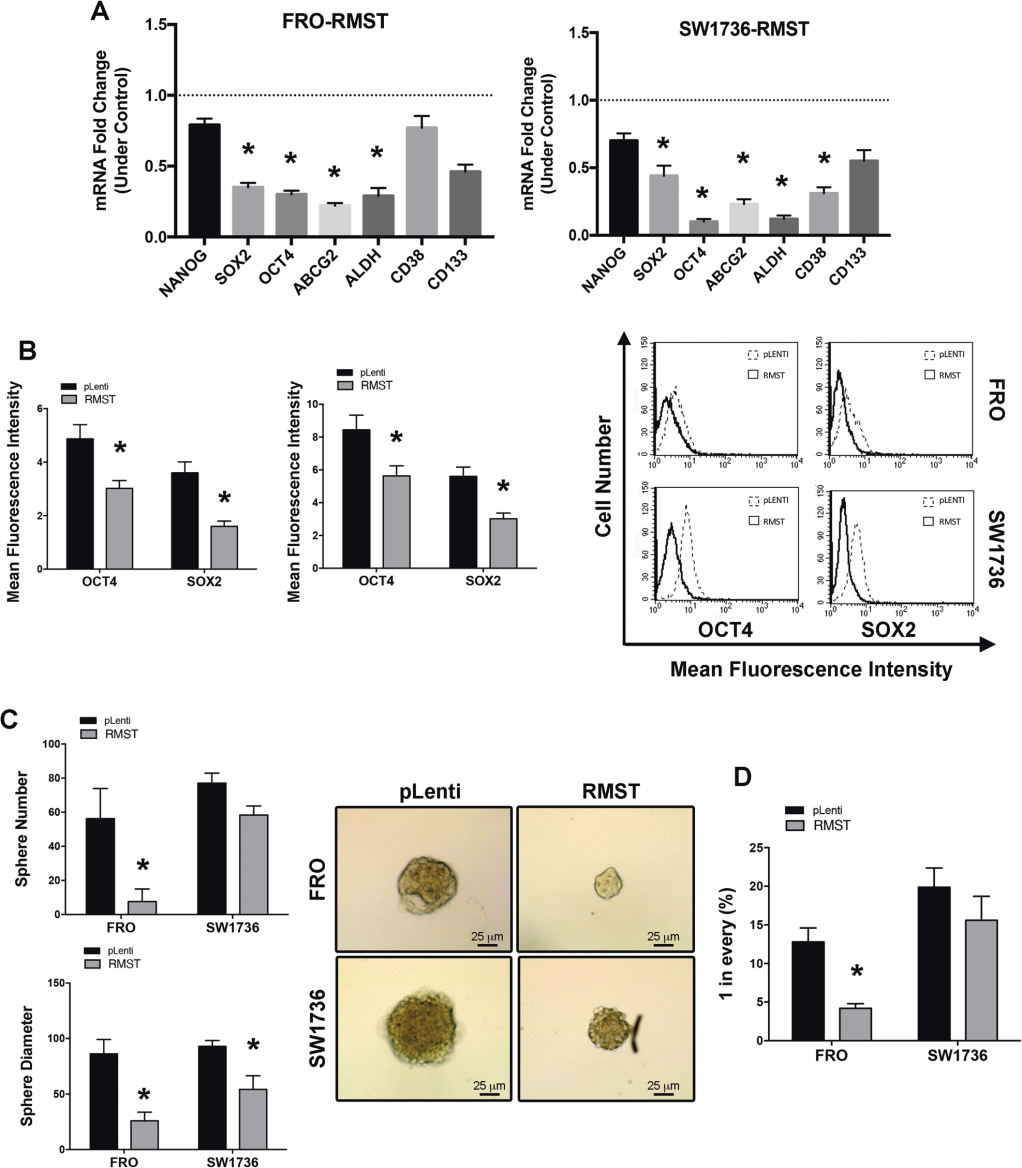
*RMST* expression modulates the stemness features of ATC cells. **A** qRT-PCR evaluation of a panel of stemness markers in FRO-*RMST* cells (left panel) and SW1736-*RMST* (right panel). Data were compared to each control cells (FRO-pLenti and SW1736-pLenti, respectively), set equal to 1, and reported as 2^-ΔΔCt^ values ± SD. **B** Citofluorimetric assay was employed to evaluate the expression of OCT4 and SOX2 proteins in FRO-*RMST* and SW1736-*RMST* cells compared to the respective control FRO-pLenti and SW1736-pLenti cells (right panel). Representative histogram plots are also presented (left panel). Data are reported as Mean Fluorescence Intensity values ± SD of three independent determinations on three mass populations, *p < 0.05 compared to the control cells (pLenti) by Student’s *t*-test. **C** Sphere Forming Assay was performed in FRO-*RMST* and SW1736-*RMST* and relative FRO-pLenti and SW1736-pLenti control cells. Data corresponding to sphere number ± SD and sphere diameter ± SD are respectively reported (left panel). Data are representative of three independent determinations on three mass populations, *p < 0.05 com*p*ared to the control cells (pLenti) by Student’s *t*-test. Representative images of spheres obtained in ATC cell models (FRO and SW1736) expressing *RMST* or not (pLenti) are shown (right panel; magnification 100 ×). **D** Effect of *RMST* on the clonogenic potential of FRO and SW1736 spheroids. Data are representative of three independent determinations on three mass populations, *p < 0.05 compared to pLenti-TC cells.

Since *RMST* physiological and functional roles involve its ability to interact with SOX2 transcription factor [23] and an inverse regulation of *RMST* in comparison to *SOX2* levels in human and mice TC samples was observed, we investigated the levels of *SOX2* in cells with or without enforced expression of *RMST*, together with the expression of several other stemness markers (*NANOG, OCT4, ABCG2, ALDH, CD38, CD133*) since SOX2 in TC is a master regulator of cancer stem cells [25, 26]. Figure 5A shows that *RMST* overexpression in both FRO and SW1736 cells significantly reduced the mRNA of *SOX2* and of several other stemness markers, such as *NANOG, OCT4, ABCG2, ALDH, CD38* and *CD133*, as assessed by qRT-PCR. The reduction in SOX2 and OCT4 expression was then confirmed also at protein level by intracellular cytofluorimetric analysis (Fig. 5B).

Thereby, to deeper characterize the role of *RMST* on TC cells stem-like features, a sphere-forming assay (which detects CSCs by their capacity to proliferate in the absence of substrate adherence) was carried out. Interestingly, as shown in Fig. 5C, the propensity of FRO and SW1736 cells to form spheroids was reduced by *RMST* overexpression, when compared to relative controls, as assessed by the number of the thyrospheres and average sphere diameter. Furthermore, we employed a limiting dilution assay on FRO and SW1736 cells cultured as thyrospheres to evaluate the impact of *RMST* on the clonogenic potential of TC cells. *RMST* transfection significantly reduced the clonogenic potential of TC cells compared to the relative controls (Fig. 5D).

## Discussion

ATC is one of the most aggressive malignancies with a very short survival (6 months on the average). Therefore, it requires a better comprehension of the molecular mechanisms underlying its development to make an early diagnosis, prevent TC progression, put on effective therapeutic treatments, thus improving the survival of ATC patients.

LncRNAs are new emerging players in human cancers and, because of their aberrant expression in this pathology, they could represent important tools for the diagnosis and novel targets for the approach of innovative therapies. In this study we aimed at characterizing the role of the *RMST* lncRNA downregulation in ATC (as reported by our group in a recent study [10]) since several studies reported the *RMST* involvement in various biological processes and suggest an important role also in TC. Indeed, it has been reported that *RMST* (a) is deregulated in a series of human cancers [14, 15], (b) exerts an anti-tumoral role in triple-negative breast cancer [17], (c) regulates neurogenesis [23], and (d) promotes neural apoptosis through interacting with the ribonucleoprotein K (hnRNPK) and through the indirect activation of p53/mir-107 signaling pathway [30].

Firstly, we evaluated *RMST* expression in a panel of TC samples confirming its drastic downregulation in ATC, whereas just a slight reduction was observed in PTC and FTC. Interestingly, *SOX2* expression was inversely correlated with that of *RMST* in the same neoplastic tissues. Subsequently, these results were further confirmed through the analysis of two publicly available databases, thereby reinforcing the hypothesis of a critical role of *RMST* downregulation in TC progression. Interestingly, *Rmst* downregulation, associated with an increase of *Sox2* expression, was also observed in UTC induced in transgenic animals carrying a mutated form of the *BRAF* gene.

According to a recent study, the expression of *RMST* was considerably lower in adjacent NT tissues than it was in PTC tissues, and this downregulation was correlated with the TNM stage and lymph node metastases [31]. A more detailed bioinformatic analysis of thyroid neoplasms, based on publicly available datasets, reported that *RMST* was specifically downregulated in ATC [32], but, despite this, *RMST* role in thyroid carcinogenesis remains unknown.

Then, its role in TC progression was investigated by restoring *RMST* expression in FRO and SW1736 cells. *RMST* repressed the main aggressive features of ATC cells, such as cell proliferation, migration and invasion. Moreover, a reduction of several mesenchymal markers was also observed in *RMST* overexpressing ATC cells when compared to the control empty vector transfected cells, indicating the involvement of *RMST* in EMT. Noteworthy, *RMST* expression is able to repress the expression of mesenchymal markers such as *SNAIL, SLUG* and *TWIST1* [33, 34]. These markers, which are transcription factors, play a fundamental role during the transition from epithelial to mesenchymal phenotype, and their expression during this phase is due to the activation of important signaling pathways such as those driven by receptor tyrosine kinase, Wnt/β-catenin, Notch, TGF-β/BMP and Hedgehog [33, 35]. Interestingly, it has been reported that lncRNAs are able to modulate these signaling pathways that culminate in the modulation of SNAIL, SLUG and TWIST1, so it is likely that *RMST* can also regulate them by acting on their upstream regulatory pathways [33].

Noteworthy, *RMST* restoration led also to a significant reduction in the expression of the stemness markers *OCT4, SOX2* and *NANOG*. It is known from the literature that these three transcription factors play a key role in regulating the stem phenotype in several human cancers including TC [36, 37], and the observation that they are downregulated by *RMST* confirms the key role of the latter in repressing the stem characteristics of TC. Furthermore, once again, as observed in the case of mesenchymal markers, the Wnt/β-catenin, Notch and Hedgehog signaling pathways are also involved in the regulation of these stemness markers [37]. This allows us to hypothesize that *RMST* can actually interfere with one of these three pathways in order to repress the expression of either aforementioned mesenchymal or stemness markers. Interestingly, the restored *RMST* expression is also capable of suppressing genes such as *ALDH1* and *ABCG2*, whose role has been seen to be associated with the acquisition of a stem phenotype in TC [38, 39]. Similarly, the *CD133* stem cell marker, which has been reported to characterize CSCs and to be associated with a poor prognosis in different tumor types, and *CD38* [40] are also repressed by *RMST*, thus confirming once again its involvement in the modulation of the stem phenotype [41].

To better elucidate the consequence of *RMST* expression in stem-like features, we carried out a sphere forming assay in FRO and SW1736 cells. Interestingly, thyrospheres deriving from *RMST*-TC cells were fewer and smaller than those deriving from pLenti-TC cells. Overall, these findings highlighting the involvement of *RMST* in the sphere formation, self-renewal and tumor-initiating ability of thyroid CSCs, may provide novel therapeutic strategies for the cure of ATC patients.

It is worth to note that the analysis of *RMST* expression in adult tissues (Fig. 1A) reveals that *RMST* is highly expressed in thyroid, whereas its expression is low or undetectable in other tissues. This would suggest a critical role of *RMST* in thyroid specific activities, likely in the appearance of thyroid differentiation. This hypothesis would be consistent with the loss of its expression in UTC samples. Experiments aimed at silencing *RMST* in thyroid differentiated cells might elucidate the role of *RMST* in thyroid differentiation. Moreover, the generation of *Rmst* knock-out mice would give important insights on the *RMST* role in thyroid development.

In conclusion, the data shown in this study support the involvement of *RMST* downregulation in the loss of differentiation and the gain of malignancy in thyroid carcinomas.

## Materials and methods

### Microarray dataset

The gene expression datasets GSE65144 and GSE33630 have been derived from GPL570 Platform (Human Genome U133 Plus 2.0 Array, Affymetrix, Santa Clara, CA, USA) (http://www.ncbi.nlm.nih.gov/geo/). The GSE65144 contains 45 NT and 11 ATC samples, whereas the GSE33630 contains 13 NT and 12 ATC samples. Deregulated lncRNAs were filtered using GEO2R (http://www.ncbi.nlm.nih.gov/geo/geo2r/), a tool used for identifying differentially expressed genes. LncRNAs with FDR adjusted *p* ≤ 0.05 and fold change (FC) ≥ 1.2 were considered being differentially expressed. Venn diagrams of deregulated lncRNAs were generated using FunRich 2.1.2 software.

### Human thyroid tissue samples

The Service d’Anatomie et Cytologie Pathologiques, Centre de Biologie Sud, Groupement Hospitalier Lyon Sud, Pierre Bénite (France), provided samples of both healthy and malignant human thyroid tissue. All patients provided their informed consent. Under the reference number DC-2011-1437, the Ministry of Research, the South-East IV People’s Protection Committee, and the Health Regional Agency were notified of the activity of conserving biological material. The Ministry of Health approved the biological material cession operation and assigned it the reference number AC-2013-1867.

### RNA extraction and quantitative Real-Time PCR (qRT-PCR)

Using Trizol reagent (Life Technologies, Grand Island, NY, USA), total RNA was isolated from cell lines and normal or malignant thyroid tissues. The QuantiTect Reverse Transcription Kit (Qiagen, Hilden, Germany) was used to generate double stranded cDNA from 1 µg of total RNA from each sample. Quantitative Real-Time PCR (qRT-PCR) experiments were carried out using CFX96 thermocycler (Bio-Rad, Hercules, CA, USA). Each PCR reaction contained 10 µl of 2 × Sybr Green (Bio-Rad), 200 nM of each primer, and 20 ng of the previously produced cDNA. The Primer-BLAST program was used to design the oligonucleotides for qRT-PCR (Table 1), which were purchased from Integrated DNA Technologies (San Diego, CA, USA). The relative gene expression was determined using the comparative C(T) technique, as previously discussed [42].

**Table 1:** List of primers.

Human gene	Sequence (5'-->3')
RMST-Fw	gcccagcccatttcattcac
RMST-Rv	gctctgtcgttccaccttga
GAPDH-Fw	agccacatcgctcagacac
GAPDH-Rv	gcccaatacgaccaaatcc
SOX2-Fw	gcgaaccatctctgtggtct
SOX2-Rv	aaaatggaaagttgggatcg
NANOG-Fw	tacctcagcctccagcagat
NANOG-Rv	ttgctattcttcggccagtt
ABCG2-Fw	tggatttacggctttgcagc
ABCG2-Rv	tcctgttgcattgagtcctgg
OCT4-Fw	agcaaaacccggaggagt
OCT4-Rv	ccacatcggcctgtgtatatc
SLUG-Fw	ccttcctggtcaagaagcat
SLUG-Rv	cacagtgatggggctgtatg
ALDH-Fw	gcaactgaggaggagctctg
ALDH-Rv	ttcgattaaatcagccaacttgt
CD133-Fw	tggtgcagaacttgacaacgt
CD133-Rv	atacctgctacgacagtcgtg
CD38-Fw	tcagccactatgaagttggga
CD38-Rv	ctggacctgtgtgaactgatgg
NES1-Fw	gctgcgggctactgaaaag
NES1-Rv	ctgccgaccttccaggac
TWIST1-Fw	ccggagcctagatgtcattg
TWIST1-Rv	ggcctgtctgcgtttctctt
βACTIN-Fw	tgcgtgacattaaggagaag
βACTIN-Rv	gctcgtagctcttctcca

Mouse gene	Sequence (5'-- > 3')
Rmst-Fw	atggcaagcctgtagagctg
Rmst-Rv	ggtgggtgatgtgaagcata
Sox2-Fw	tccaaaaactaatcacaacaatcg
Sox2-Rv	gaagtgcaattgggatgaaaa
Gapdh-Fw	tgcaccaccaactgcttagc
Gapdh-Rv	tcttctgggtggcagtgatg

### Cell culture, transfections and spheroid-forming assay

The Dulbecco’s modified Eagle’s medium (DMEM; Sigma-Aldrich, St. Louis, MO, USA) was supplemented with 10% fetal bovine serum (Euroclone, Milan, Italy), 1% penicillin/streptomycin and 1% L-glutamine (Sigma-Aldrich) to grow the FRO and SW1736 cancer cell lines. Cells were kept at 37 °C in a 5% CO_2_ environment. Cell lines were routinely tested for the presence of mycoplasma contamination by using EZ-PCR Mycoplasma Detection Kit (Biological Industries, Beit-Haemek, Israel).

In accordance with the manufacturer’s instructions, cells were transfected using Life Technologies’ Lipofectamine 2000 reagent. Applied Biological Materials Inc. (Ferndale, WA, USA) sold the human RMST expression plasmid. qRT-PCR testing verified RMST expression. 1.5 µg/ml of puromycin (Life Technologies) was used to generate the FRO and SW1736 stable-transfected cells.

Cells were plated in serum-free DMEM/Nutrient Mixture F-12 Ham (DMEM/F12 = 1:1, Life Technologies) enriched with 2% B27, 10 ng/ml of epidermal growth factor and 20 ng/ml of basic fibroblast growth factor (Miltenyi Biotec, Bergisch Gladbach, Germany), at a density of 7 cells per well on ultra-low-attachment 96-well (Corning Incorporated, Painted Post, NY, USA), for the spheroid-forming test. Cells were kept at 37 °C in a humidified 5% CO_2_ environment. After 14 days spheres were counted. The formula (number of developed spheres/number of wells carrying cells) × 100 was used to calculate the number of spheres [28]. Cells were dropped at 1, 5, 10, and 50 cells/well for limiting dilution tests. Statistical significance and clonal frequency were assessed using the Extreme Limiting Dilution Analysis ‘limdil’ tool (http://bioinf.wehi.edu.au/software/elda/index.html) [28].

### Cell migration, invasion and scratch assays

Transwell migration and invasion tests were carried out in Transwell chambers (8-μm pores; Euroclone). Matrigel (Sigma-Aldrich) was applied to the upper side of the Transwell chamber for the invasion assay. In summary, serum-deprived media was used to plate FRO and SW1736 cells (3 × 10^4^ or 6 × 10^4^ cells for migration and invasion tests, respectively) on the upper side of the Transwell chamber. A volume corresponding to 300 μl of complete medium was then delivered to the inferior chamber as a chemoattractant. After 24 h, a crystal violet solution (crystal violet 0.05%, methanol 20%) was used to stain the cells that had moved to the lower side of the chambers. The invasion assay used the same approach after 48 h. FRO and SW1736 cells were properly seeded in a 96-well plate to standardize the cell count. After 4 h, the absorbance at 490 nm was measured using a microplate reader (LX800, Universal Microplate Reader, BioTek Instruments, Inc., Winooski, VT, USA) using a CellTiter assay (Promega Corporation, Madison, WI, USA). The chamber’s crystal violet was then de-stained with a PBS-0.1% SDS solution before being read at 570 nm. By normalizing the values of crystal violet to those of CellTiter, the results were obtained.

Confluent TC cell monolayers were treated with mitomycin (2 μg/ml for 2 h) (Sigma-Aldrich) for wound-healing tests. With the use of a p10 pipet tip, a "scratch" was made on the cell monolayer. For 24 h, cells were permitted to migrate at 37 °C in an incubator. A phase-contrast microscope was used to acquire multiple photograms, which were then quantitatively analyzed using specialized software (Image J, National Institutes of Health, Bethesda, MD, USA) [28]. The wound closure rate was estimated by comparing the wound width (WW) at time 0 (t0) and time 24 (t24), respectively. Wound closure rate is calculated as (WWt0-WWt24) RMST/(WWt0-WWt24) pLenti × 100.

### Cytofluorimetric analysis

Cells were treated with isotype control or specific antibodies for 30 min at 4 °C. Cell Signaling (Beverly, Massachusetts, USA) provided the human SOX2 antibody, while the anti-OCT4-PE antibody came from Miltenyi Biotec [43]. CellQuest software was used to evaluate cells employing a FACS Calibur cytofluorimeter (BD Biosciences, Franklin Lakes, NJ, USA; www.bdbiosciences.com). Each sample covered at least 10^4^ events that were recorded. Cells were permeabilized when required using the Cytofix/Cytoperm kit from BD Biosciences [28].

Cells were permeabilized with cold 70% ethanol in PBS for cell cycle analysis and stored at −20 °C until next day. Cells were then resuspended in PBS supplemented with 50 μg/ml propidium iodide (PI) and 100 mg/ml RNase, and were kept for 30 min at 30 °C. CellQuest software was used to analyze the data obtained through the FACS Calibur [28].

Dead cells were identified and distinguished using the Annexin V-FITC Kit from Miltenyi Biotec. Cell staining was performed in a dark environment for 15 min at room temperature by adding 10 μl of Annexin V-FITC to 10^6^ cells, following a serum starvation of 18 h. Prior to analysis employing CellQuest software and the FACS Calibur, 5 μl of PI solution was added [28].

### Mouse model

Tg-rtTA/TetO *BRAF*^V600E^ double transgenic mice were kept in the University Federico II’s (Naples, Italy) animal facility, which has received approval from the Italian Ministry (D.M. 78/213-A and 12/2018-UT), under pathogen-free conditions with controlled humidity, light and temperature. They were also given access to standard food and water as well as 2500 mg/kg of doxycycline for 1 week. According to the 3Rs principle and in order to minimize the mouse sample number, we employed four (*n* = 4) mice either for the control and for the treated group, achieving statistically significant differences. No randomization and blinding criteria were applied. The Institutional Animal Care and Use Committee (IACUC) of University Federico II and the Italian Ministry of Health have both given their approval for the study, which has 2013/0078506 as protocol number.

### Immunohistochemistry

Thyroids were embedded in paraffin after being fixed in 4% PFA and dehydrated. From paraffin blocks, sections 7 μm-thick were cut, then being processed for the subsequent analysis. The sections were permeabilized for 5 min with PBS-0.2% triton and then washed with PBS twice for 5 min. Subsequently, they were put in the microwave for 15 min in citrate buffer (0.01 M, pH6) for antigen unmasking. Endogenous peroxidases were subsequently blocked with 1.5% oxygen peroxide and methanol and tissues were made permeable with PBS-0.2% triton for 5 min, washed with PBS twice for 5 min, and then blocked at room temperature for 1 h with a PBS solution (blocking solution) consisting of 3% BSA, 20 mM MgCl_2_, 0.3% tween 20, and 5% normal goat serum (S-1000, Vector Laboratories, Newark, CA, USA). Incubation with primary antibodies was performed at 4 °C overnight in blocking solution. Sections were then washed for 5 min with PBS-0.2% triton and with PBS twice for 5 min and subsequently incubated at room temperature for 1 h with a fresh blocking solution containing 1:100 secondary antibody (biotinylated anti-rabbit and anti-mouse IgG (H + L); Vector Laboratories). After secondary antibodies incubation and washing (5 min with PBS-0.2% triton and twice for 5 min with PBS), section were incubated for 30 min at room temperature with ABC (SK-4000, Vector Laboratories). Finally, DAB substrate (SK-4100, Vector Laboratories) was added after washing with PBS-0.2% triton for 5 min and thrice for 5 min with PBS. After staining with eosin (Surgipath 3801602E, Leica Biosystems, Wetzlar, Germany), sections were de-hydrated, treated with DPX Mountant (GRM655, HiMedia Laboratories, Mumbai, Maharashtra, India) and covered with glasses. Axioskop microscope equipped with an Axiocam 105 color digital camera (Zeiss, Oberkochen, Germany) was employed to acquire images, that were subsequently analyzed with the Axio Vision software [22].

### Statistical analysis

GraphPad Prism was utilized to conduct the statistical evaluations. *T*-test and Anova test were employed to assess the collected data’s statistical significance. We used the Bonferroni post-test to evaluate group differences when the Anova test was significant (*p* < 0.05). In each experiment, the significance was evaluated at *p* < 0.05. The mean values and standard deviation (SD) of the data are presented. The cBioPortal database was used to depict clinical-pathological characteristics with respect to *RMST* expression levels [44].

## Supplementary information

Supplementary figure legends

Supplementary Figure 1

Supplementary Figure 2

Supplementary Figure 3

## Data Availability

All data generated during this study are included in this published article and in its supplementary information files. Confirmatory datasets analyzed in this study are freely publicly available and their specifications are indicated along the manuscript.
