# Supplementary material for: The lncRNA RMST is drastically downregulated in anaplastic thyroid carcinomas where exerts a tumor suppressor activity impairing epithelial-mesenchymal transition and stemness
Source: Cell Death Discov. 2023 Jul 1;9:216. doi: 10.1038/s41420-023-01514-x (PMC10314909; doi:10.1038/s41420-023-01514-x)
Supplement: Supplementary file 1 — Supplementary figure legends [file 41420_2023_1514_MOESM1_ESM.docx]

**Supplementary figure legends.**

**Supplementary Fig. 1. *RMST* correlation to TC patients’ characteristics.** *RMST* mRNA expression levels of 500 patients affected by TC stratified for overall survival status and disease- and progression-free status.

**Supplementary Fig. 2. *RMST* effects on TC cell cycle.** Cell cycle distribution of FRO **(A)** and SW1736 **(B)** cells transfected with *RMST* or the relative empty vector (pLenti), after 18 h of serum deprivation, measured by propidium iodide (PI) staining and flow cytometry. The percentage of the cells distributed in G1, S, G2/M phases is shown.

**Supplementary Fig. 3. *RMST* effects on TC cell morphology.** Morphology of FRO **(A)** and SW1736 **(B)** cells transfected with *RMST* or the empty vector (pLenti). Multiple photograms are shown (magnification 100×).
