# Supplementary figures and images for: The lncRNA RMST is drastically downregulated in anaplastic thyroid carcinomas where exerts a tumor suppressor activity impairing epithelial-mesenchymal transition and stemness
Source: Cell Death Discov. 2023 Jul 1;9:216. doi: 10.1038/s41420-023-01514-x (PMC10314909; doi:10.1038/s41420-023-01514-x)

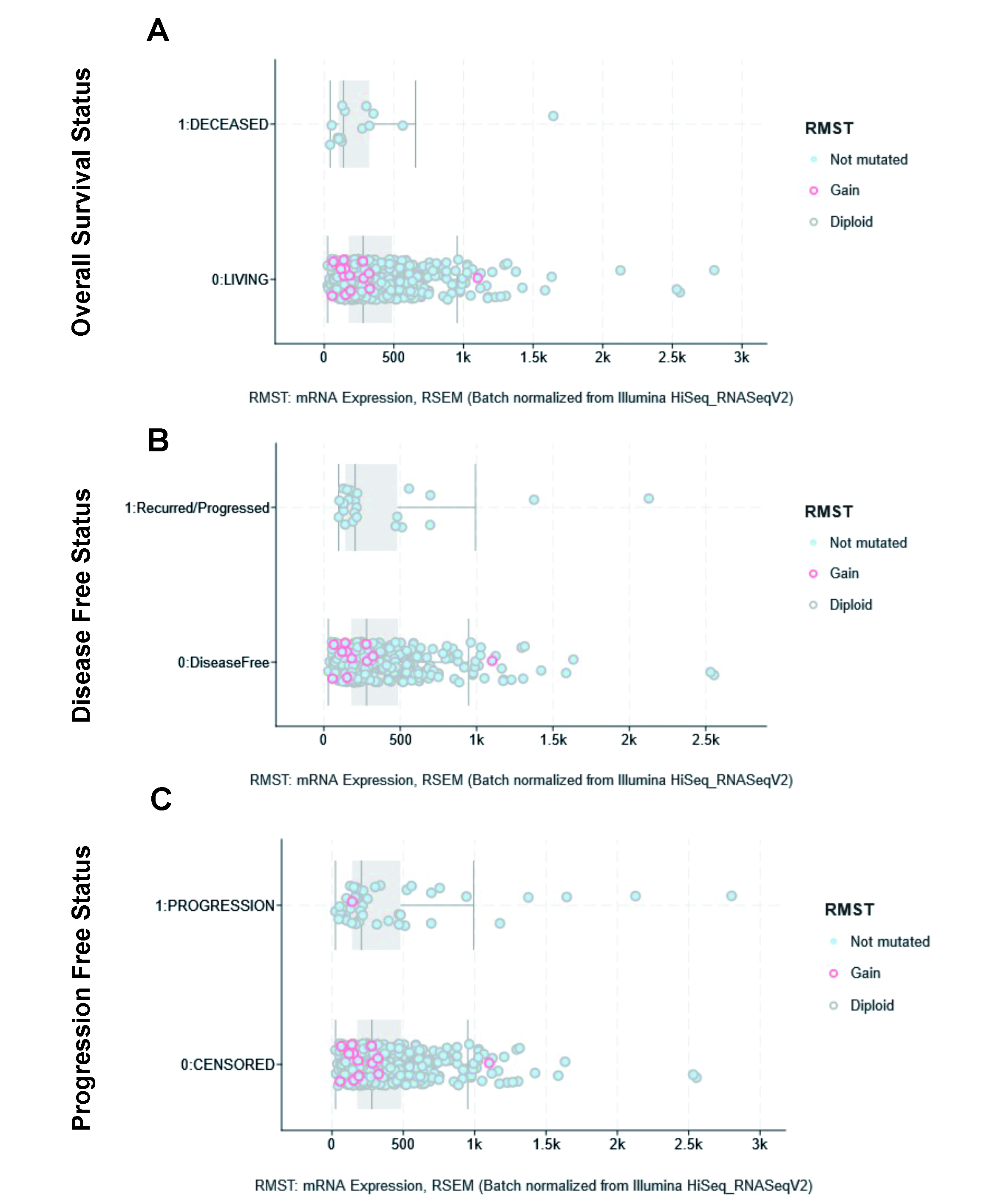

Supplement: Supplementary file 2 — Supplementary Figure 1 [file 41420_2023_1514_MOESM2_ESM.tif]

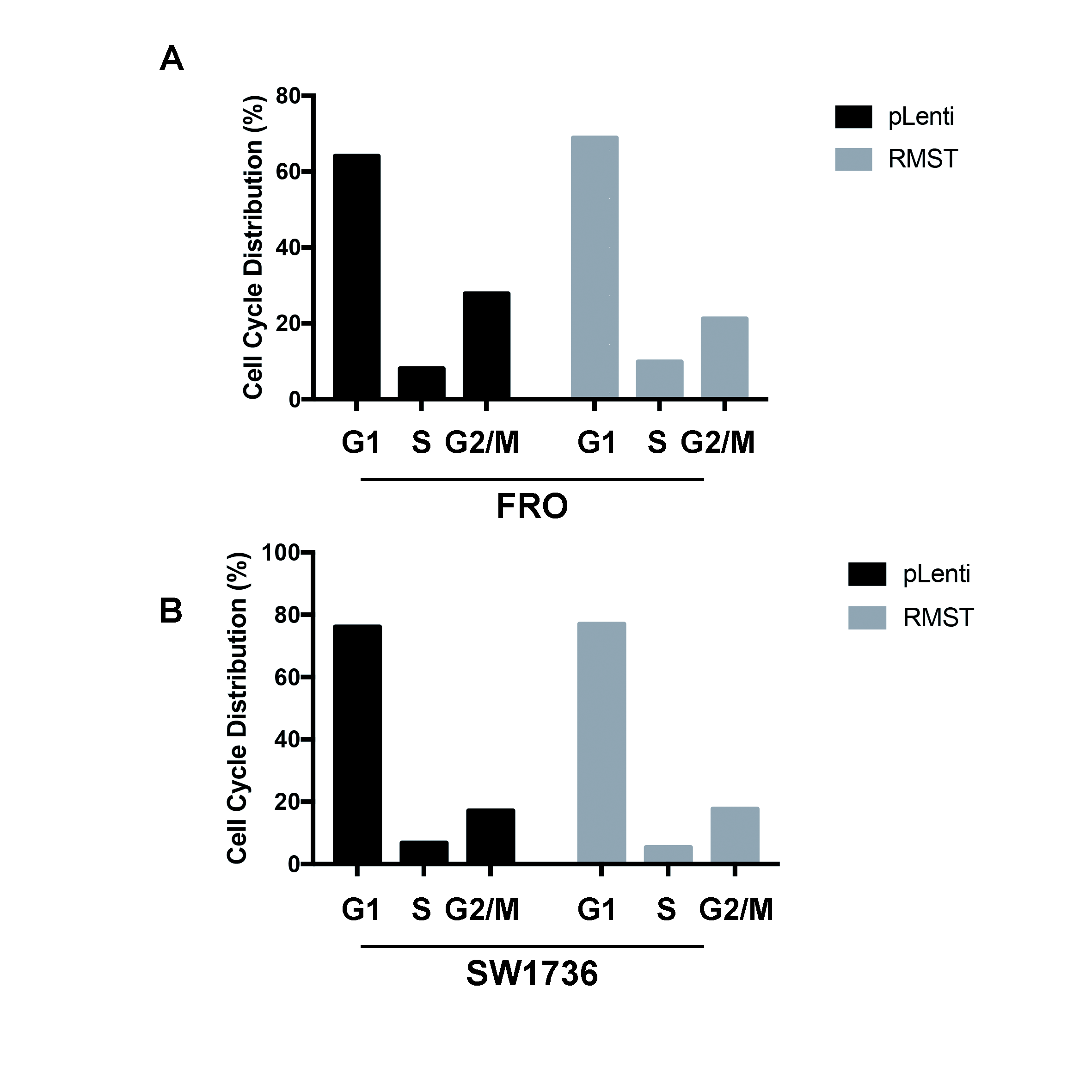

Supplement: Supplementary file 3 — Supplementary Figure 2 [file 41420_2023_1514_MOESM3_ESM.tif]

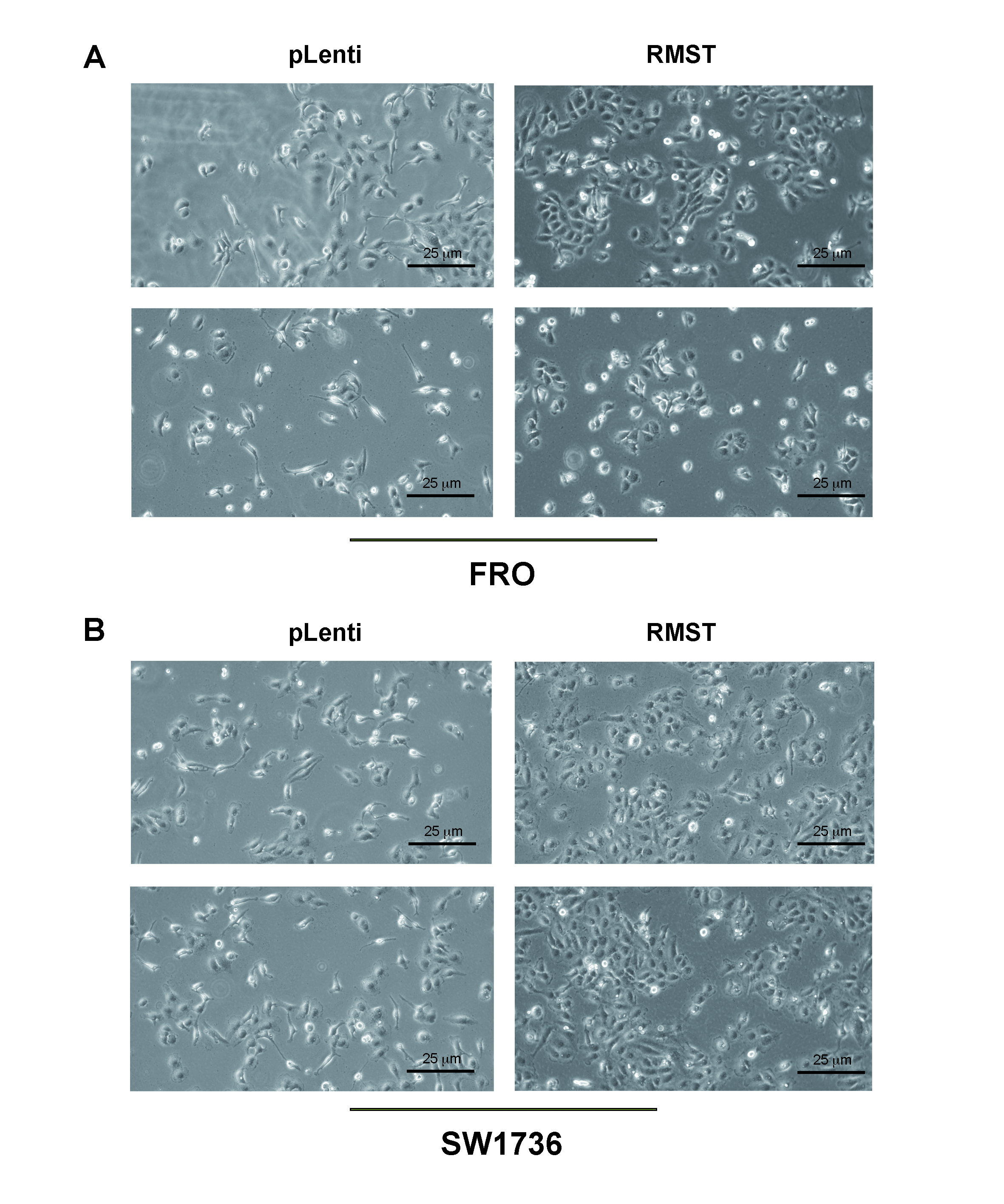

Supplement: Supplementary file 4 — Supplementary Figure 3 [file 41420_2023_1514_MOESM4_ESM.tif]
